# Editorial: Reducing the Mortality Gap in People With Severe Mental Disorders: The Role of Lifestyle Psychosocial Interventions

**DOI:** 10.3389/fpsyt.2019.00434

**Published:** 2019-06-20

**Authors:** Andrea Fiorillo, Mario Luciano, Maurizio Pompili, Norman Sartorius

**Affiliations:** ^1^Department of Psychiatry, University of Campania “L. Vanvitelli”, Naples, Italy; ^2^Department of Neurosciences, Mental Health and Sensory Organs, Suicide Prevention Center, Sant’Andrea Hospital, Sapienza University, Rome, Italy; ^3^Association for the Improvement of Mental Health Programs, Geneva, Switzerland

**Keywords:** excess mortality, comorbidity, severe mental disorders, psychosocial interventions, healthy behaviors

Compared to other non-communicable diseases, severe mental disorders (SMDs)—namely major depression, bipolar disorders, schizophrenia, and related spectrum disorders—are associated with a larger morbidity, poorer health outcomes, and higher mortality rates (1, 2). People suffering from SMD die on average 10 to 20 years earlier than the general population (3–4), and this gap tends to increase over time (5). Only a minority of premature deaths are attributable to unnatural causes, such as suicide, homicides, or accidents (6), while the vast majority of deaths is due to physical health problems, such as cardiovascular, respiratory or infectious diseases, diabetes mellitus, and cancers. This is why the excess mortality in people with SMD has been defined as a “public health scandal” (4).

Excess mortality and morbidity in SMD can be explained by intertwined components, including patients’ individual factors (e.g., severity of symptoms, impulsivity, emotional dysregulation, deficits in cognitive and social skills), lifestyle behaviors (e.g., smoking, poor diet, sedentary behavior, alcohol and drug abuse), social disadvantages of people with SMDs (i.e., stigma, discriminating policies, unemployment, homelessness, limited family, social, and community resources), and healthcare disparities (e.g., poor quality of service provision, limited access to health information, reduced prescriptions for physical checkups, professionals’ negative attitudes towards people with mental disorders) (7).

In the recent years, it became clear that the management of physical illnesses in people with SMD is challenging, and it is likely to become even more problematic in the years to come (8) due to several factors, including: 1) the extension of life expectancy of all members of the population, due to medical advancements, improvements in hygiene and food supply, and the consequent increase in the probability of developing physical disorders, particularly the noncommunicable ones (such as, cancer, diabetes, cardiovascular illness) (9); 2) the over-division and over-specialization of medical disciplines and the fragmentation of medical knowledge (10), which has also occurred within the field of psychiatry, with the consequence that in some cases psychiatrists deal only with specific age groups (children, adolescents, elderly) or specific diseases (e.g., only with patients with bipolar or eating disorders) or stages in disease-development (e.g., early intervention specialists) or treatment procedures; and 3) the increasing gap in knowledge within disciplines due to refinement and extension of training (e.g., between nurse assistants, nurses, specialized nurses, and PhD nurses) and the continuing lack of awareness of the need to prepare health workers during their training to deal with comorbidity.

In order to address this topic, the World Health Organization, within the Global Action Plan for the Prevention and Control of non-communicable diseases, has provided a set of actions to be undertaken by national health systems, including control of risk factors, scaling up management in primary health care, and development of national policies (11).

As awareness of this problem rises, it becomes clear that a single directed approach would not be enough, as excess mortality cannot be attributable to a single factor; therefore, a multilevel approach, in which the different stakeholders involved in health care provision establish workforces for the long-term management of physical and mental health conditions, is recommended (12). The different stakeholders would include policy makers, psychiatrists, other medical specialists, users, and carers of people with mental disorders (13).

Interventions aimed at reducing risk factors and improving physical health of people with SMDs may act at different levels, including: 1) the community and policy-making levels, through the development of national policies for the promotion of healthy lifestyle behaviors and the development of comprehensive health-care packages; 2) the health-system organizational level, through the improvement of screening programs for physical conditions, the promotion of care coordination strategies, and the development of guidelines for an integrated delivery of mental and physical health care; and 3) the individual clinical level, through the promotion of early management and treatment of physical conditions in patients with SMDs, the development, and the implementation of lifestyle behavioral psychosocial interventions addressing weight loss, tobacco smoking, healthy diet, physical exercise, and risky sexual behaviors (7).

In the Research Topic “Reducing the Mortality Gap in People With Severe Mental Disorders: The Role of Lifestyle Psychosocial Interventions,” we have addressed the individual clinical level. In particular, the aim of our research topic was to summarize the current knowledge on the efficacy and effectiveness of psychosocial interventions aimed at improving lifestyle behaviors and at promoting physical health in patients with SMDs. Recently, several interventions targeting lifestyle behaviors have been developed, and many of them have proven to be effective both in randomized controlled trials and in routine clinical practice, although their dissemination on a large scale has not been satisfying so far (14). Other concerns are related to the following: a) The geographical distribution of available findings. In fact, national data about mortality of people with SMDs and about the efficacy of interventions to promote healthy habits in low- and middle-income countries are very scarce, particularly in those countries where mortality rates for infectious diseases and the reduced availability of healthcare resources could be responsible of an even greater morbidity and mortality of people with SMDs (15); b) the duration and intensity of psychosocial treatments, with the need of cost-effectiveness analysis; and c) the identification of effective delivery packages in routine care. In fact, these interventions can be delivered either individually or in group settings, with little evidence supporting differential efficacy profiles among the different formats.

Our research topic includes seven original research papers, three brief research reports, three perspective papers, one opinion paper, and four review papers. In particular, the 18 papers have focused on two main areas: a) the clinical characterization of patients with SMDs at risk of developing physical illnesses; and b) the different psychosocial strategies for the management of physical comorbidities in patients with SMDs.

As regards the clinical characterization of patients with SMD at risk of developing physical health problems, Ventriglio et al. have investigated the effect of oral vs. long-acting injectable antipsychotics on the onset of metabolic syndrome; Cuomo et al. have explored the possible relationship between smoking habits and vitamin D deficiency, while Bartoli et al. in their review have pointed out that the presence of depressive symptoms in patients with stroke is associated with higher mortality rates compared to those non-affected by post-stroke depression. The relationship between the presence of medical unexplained physical symptoms and the occurrence of mental disorders has been explored by Poloni et al. in a sample of 5,039 patients. Finally, the relationship between lifestyle behaviors, mental health, and suicide risk has been deepened by Berardelli et al. in their narrative review.

As regards the role of psychosocial interventions in the management of physical comorbidities in patients with SMDs, the papers by Dalcin et al. and Herbsleb et al. have dealt with the reduction of cardiovascular risk factors, while Elkholy et al. and Burns et al. have promoted interventions to reduce tobacco smoking in these patients. Interventions to promote physical exercise (Schmitt et al.; Belvederi Murri et al.; Korman et al.) and weight loss (McGinty et al.) have been described and their efficacy has been tested. Many psychosocial approaches have been developed to deal with more than one lifestyle behavior (e.g., Sampogna et al.; Taylor et al.; Kuzman et al.; Berardelli et al.; Barber and Thornicroft), while the role of carers and family members in promoting patients’ healthy behaviors has been highlighted in the paper by Onwumere et al.

Taken together, all papers included in this Research Topic provide new insights on the topic of comorbidity between mental and physical disorders, highlighting that several efforts have already been made in this direction, but much work still remains to be done. In the years to come, research should focus on the identification of protective factors that could reduce morbidity and mortality of people with SMDs, on the identification of comorbid substance abuse, on the investigation of cost-effectiveness of psychosocial interventions, and on the identification of the characteristics of the interventions that can positively impact on patients’ behaviors.

## Author Contributions

All authors listed have made substantial, direct, and intellectual contribution to the work and approved it for publication.

## Conflict of Interest Statement

The authors declare that the research was conducted in the absence of any commercial or financial relationships that could be construed as a potential conflict of interest.
